# Cardiac Point-of-Care Ultrasound Performed in a Stroke Unit Is Associated with a Reduced Hospital Length of Stay

**DOI:** 10.3390/jcm15051885

**Published:** 2026-03-01

**Authors:** María Luisa Ruiz-Franco, Rodrigo José Milán-Pinilla, Laura Amaya-Pascasio, Antonio Arjona-Padillo, Manuel Payán-Ortíz, María Victoria Mejías-Olmedo, Javier Fernández-Pérez, Patricia Martínez-Sánchez

**Affiliations:** 1 Department of Neurology, Torrecardenas University Hospital, 04009 Almería, Spainaarjonap@gmail.com (A.A.-P.);; 2 Faculty of Health Sciences, CEINSA Research Centre, University of Almeria, 04120 Almería, Spain

**Keywords:** stroke, stroke unit, cardiac point-of-care ultrasound (cPOCUS), echocardiogram, length of stay

## Abstract

**Objectives:** Cardiac point-of-care ultrasound (cPOCUS) enables rapid bedside cardiac assessment and may facilitate early identification of potential cardiac sources of embolism in patients with acute ischemic stroke (AIS). This study aimed to evaluate whether neurologist-performed cPOCUS is associated with reduced hospital length of stay (LOS) in patients admitted to a Stroke Unit (SU). **Methods**: We conducted a retrospective observational study including consecutive patients with AIS admitted between 2020 and 2021 who required cardiac ultrasound for etiological evaluation. Patients underwent cPOCUS and/or transthoracic echocardiography (TTE) and were classified into two groups: those evaluated with cPOCUS (with or without TTE) and those evaluated exclusively with TTE (control group). The availability of cPOCUS depended on predefined weekly schedules rather than individual clinical decision-making, partially mitigating selection bias. The primary outcome was LOS. Multivariable linear regression analysis was performed to adjust for potential confounders. **Results**: Among 808 patients with AIS, 332 underwent cardiac ultrasonography during hospitalization: 219 in the cPOCUS group and 113 in the control group. Overall, 60.4% were male, the mean age was 68.4 years (SD 13.3), and the median National Institutes of Health Stroke Scale score at admission was 5 (IQR 9), with no significant differences between groups. Median LOS was shorter in the cPOCUS group than in the control group [7 days (IQR 4) vs. 8 days (IQR 5); *p* = 0.015]. After adjustment for confounders, cPOCUS evaluation remained independently associated with shorter LOS (β −1.49, standard error 0.73, 95% CI −2.93 to −0.05; *p* = 0.04). **Conclusions**: Neurologist-performed cPOCUS is independently associated with reduced LOS in patients with AIS admitted to an SU. These findings suggest that cPOCUS may facilitate more efficient in-hospital workflows and support its potential integration into routine stroke care pathways.

## 1. Introduction

Cardioembolic stroke is the etiological subtype associated with the highest burden of disability and accounts for up to 30% of all ischemic strokes [1,2]. Its diagnostic evaluation relies on systematic screening for potential embolic cardiac sources, in which transthoracic echocardiography (TTE) plays a central role [3]. However, TTE often requires cardiology involvement, which may delay etiological assessment during acute stroke care and prolong in-hospital workflows, potentially impacting hospital length of stay [4].

Cardiac point-of-care ultrasound (cPOCUS) is a focused cardiac ultrasound examination performed by non-cardiologist physicians to address specific clinical questions. Unlike the comprehensive and regulated approach of TTE, cPOCUS is problem-oriented and based on targeted two-dimensional and color Doppler imaging. While standard echocardiography includes transthoracic, transesophageal, and contrast-enhanced modalities, cPOCUS is performed exclusively via the transthoracic approach and is intended to provide a rapid, suggestive assessment rather than a definitive diagnosis [2,4].

In the neurological setting, cPOCUS can be used to screen for embolic cardiac diseases and may be performed by neurologists following structured training. This technique enables the early identification of relevant structural and functional cardiac abnormalities and has the potential to accelerate etiological classification and subsequent management decisions without increasing the burden on other departments [2,4]. Although cPOCUS protocols vary across organizations, there is general consensus regarding their core components [5,6,7,8,9,10]. Standard protocols typically include parasternal, apical, subcostal, and supraclavicular views using a sector transducer [6,11].

In recent years, neurologist-performed cPOCUS has been increasingly implemented in Stroke Units, where it may expedite cardiac workup and favorably impact length of stay by enabling earlier etiological assessment and clinical decision-making. However, despite growing evidence supporting the diagnostic feasibility and accuracy of cPOCUS in stroke populations, its impact on healthcare process outcomes—particularly hospital length of stay (LOS)—has not been systematically evaluated. LOS is a clinically relevant metric in stroke care, as it reflects in-hospital workflow efficiency, resource utilization, risk of complications, and healthcare costs. In healthcare systems with limited access to formal echocardiography, delays in cardiac evaluation may prolong hospitalization even when patients are otherwise clinically stable for discharge. Therefore, understanding whether neurologist-performed cPOCUS is associated with shorter LOS addresses an important organizational and healthcare delivery knowledge gap. In this study, we assessed the effect of implementing neurologist-performed cPOCUS on length of stay among patients admitted to a Stroke Unit (SU).

## 2. Methods

### 2.1. Study Design and Population

We conducted a retrospective observational study including consecutive patients admitted with a diagnosis of acute ischemic stroke (AIS) between January 2020 and December 2021 to the Stroke Unit of Torrecárdenas University Hospital, Almería, Spain, a tertiary care center serving a reference population of approximately 760,000 inhabitants. Patients were eligible if they required cardiac ultrasonographic evaluation as part of the etiological work-up of cerebrovascular disease.

### 2.2. Cardiac Imaging Strategy and Study Groups

In patients with ischemic stroke requiring screening for embolic cardiac sources, TTE was requested from the Cardiology Department according to standard clinical practice. In parallel, all patients underwent a scheduled neurosonological evaluation performed by the Neurology Department; on one predefined day per week, the examination was conducted by a neurologist trained in cardiac point-of-care ultrasound (cPOCUS). Consequently, patients admitted or evaluated during these sessions underwent initial cardiac screening with cPOCUS, whereas patients assessed on other days followed the conventional diagnostic pathway with TTE alone.

If cPOCUS revealed no relevant cardiac abnormalities, no further echocardiographic studies were performed. In cases with equivocal findings—such as suspected ventricular dysfunction, valvular abnormalities, or intracardiac masses—and in all patients who did not undergo cPOCUS, TTE was subsequently performed. When TTE was performed as the initial examination, cPOCUS was not carried out.

Patients were classified into two groups: (1) the cPOCUS group, comprising patients who underwent neurologist-performed cPOCUS screening, regardless of whether a TTE was also performed; and (2) the control group, comprising patients who underwent TTE exclusively.

Although allocation to cPOCUS depended on predefined weekly schedules rather than individual clinical judgment, this approach does not fully eliminate potential selection bias. Admission timing, stroke severity fluctuations across days, staffing patterns, or unmeasured workflow factors could have influenced group allocation. Therefore, residual confounding cannot be excluded.

### 2.3. Variables and Data Collection

Demographic variables included age and sex. Clinical variables comprised vascular risk factors, toxic habits, comorbidities, and stroke-related characteristics. Vascular risk factors included hypertension, dyslipidemia, and diabetes mellitus, defined by prior diagnosis and/or ongoing treatment. Toxic habits included smoking, alcohol use, and illicit drug use. Comorbidities included ischemic heart disease, prior stroke, and atrial fibrillation (pre-existing or newly diagnosed). Stroke characteristics included vascular territory (anterior vs. posterior circulation), stroke severity assessed using the National Institutes of Health Stroke Scale (NIHSS), reperfusion therapies (intravenous thrombolysis and/or mechanical thrombectomy), in-hospital complications (lower respiratory tract infection and/or urinary tract infection), and hospital length of stay.

Echocardiographic variables assessed by TTE included left ventricular ejection fraction, left ventricular hypertrophy and dilation, left atrial dilation, segmental wall motion abnormalities, intracardiac masses, and valvular disease. cPOCUS variables included left ventricular hypertrophy and dilation, left atrial dilation, global or segmental hypokinesia, intracardiac masses, and valvular abnormalities.

### 2.4. cPOCUS Protocol

cPOCUS examinations were performed using a portable ultrasound device (Vivid IQ Premium v204) equipped with a multifrequency sector probe (1.5–4.6 MHz). Standard transthoracic views were obtained following focused cardiac ultrasound principles. All examinations were performed by neurologists specialized in stroke who had received structured training in cPOCUS (AA-P and LA-M). Training included formal theoretical and practical instruction in focused cardiac ultrasound according to national consensus recommendations, followed by supervised practice before independent performance.

Cardiac structure and function were assessed qualitatively. Left atrial diameter was measured in the parasternal long-axis view. Left ventricular systolic function was evaluated visually across multiple views and classified as normal, probably reduced, or severely reduced. Segmental wall motion abnormalities were assessed, and intracardiac masses were suspected based on morphology and mobility. Valvular morphology and function were evaluated using two-dimensional and color Doppler imaging.

Findings were considered “equivocal” when image quality was suboptimal or when qualitative assessment suggested possible but not definitive abnormalities, including suspected mild-to-moderate ventricular dysfunction, uncertain regional wall motion abnormalities, indeterminate valvular regurgitation severity, or doubtful intracardiac masses. In such cases, comprehensive TTE was systematically requested.

Given the retrospective design and the fact that each examination was performed by a single operator, formal inter-observer variability was not assessed. However, operators followed standardized acquisition protocols to minimize variability.

### 2.5. Statistical Analysis

Categorical variables were compared using the χ^2^ test or Fisher’s exact test, as appropriate. Continuous variables were compared using Student’s *t* test or the Mann–Whitney *U* test, depending on data distribution.

Multivariable linear regression analysis was performed to identify factors independently associated with hospital length of stay. The model included age, sex, variables previously associated with length of stay (including posterior circulation stroke and infectious complications), and variables with *p* < 0.20 in univariable analyses. A two-sided *p* value ≤ 0.05 was considered statistically significant.

Statistical analyses were performed using IBM SPSS Statistics version 27. Figures were generated using Python 3.11, with pandas for data handling and Matplotlib (version 3.8.2) for visualization.

### 2.6. Ethical Approval

The study was approved by the Provincial Research Ethics Committee of Almería (approval code 102/2022). Informed consent was waived due to the retrospective design and anonymization of data.

## 3. Results

**Figure 1:**
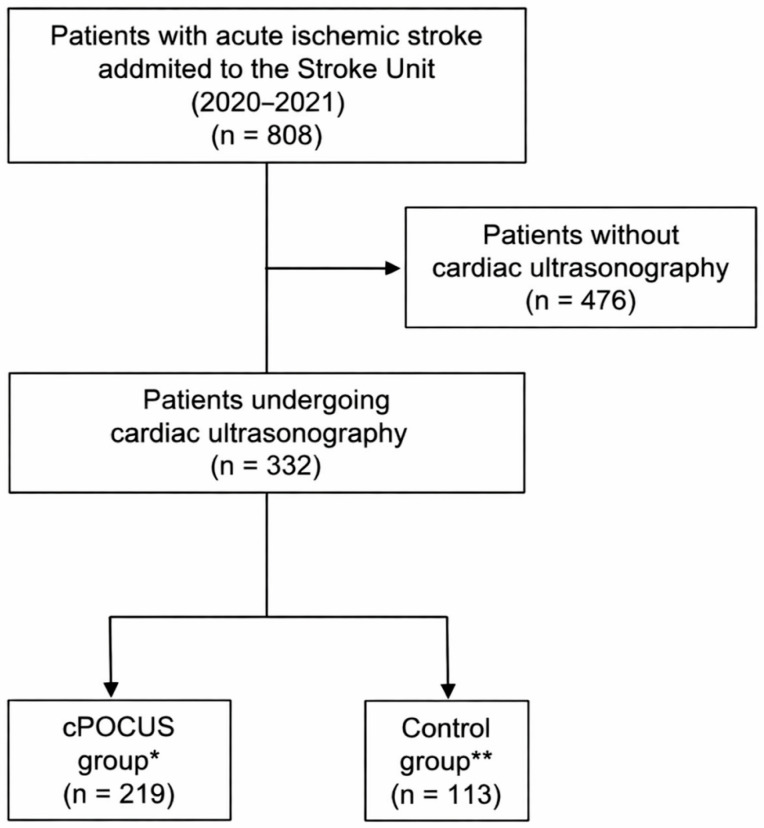
Flow diagram illustrating the inclusion of patients in the study. * cPOCUS group: Cardiac point-of-care ultrasound screening, followed or not by transthoracic echocardiography. ** Control group: transthoracic echocardiography.

A total of 808 patients with AIS were admitted to the SU during the study period (Figure 1). Cardiac imaging with cPOCUS and/or TTE was performed during hospitalization in 332 patients (39.4%). Of these, 201 (60.4%) were men, the mean age was 68.4 years (SD 13.3), and the median NIHSS score at admission was 5 (9) (Table 1).

Among the 332 patients who underwent cardiac imaging, 219 (65.9%) were assigned to the cPOCUS group and 113 (34.0%) to the control group. Baseline demographic and clinical characteristics were largely comparable between the cPOCUS and control groups (Table 1). Median age was similar in both groups (70 years in each; *p* = 0.05), as was sex distribution, with no significant differences in the proportion of women. The prevalence of major vascular risk factors, including hypertension, diabetes mellitus, atrial fibrillation, smoking, alcohol consumption, and drug use, did not differ significantly between groups. Dyslipidemia was more frequent in the cPOCUS group compared with the control group (46.6% vs. 32.7%; *p* = 0.02). NIHSS scores were comparable between groups, as was vascular territory involvement. Regarding acute treatments, the proportion of patients receiving intravenous fibrinolysis did not differ significantly, whereas mechanical thrombectomy was more frequently performed in the cPOCUS group than in the control group (24.7% vs. 14.2%; *p* = 0.02). Because mechanical thrombectomy is typically associated with more severe strokes and potentially longer hospitalization, this imbalance could have biased results against the cPOCUS group. Mechanical thrombectomy was therefore included in the multivariable regression model to adjust for its potential confounding effect. Rates of in-hospital complications, including lower respiratory tract infection and urinary tract infection, were similar between groups.

**Figure 2:**
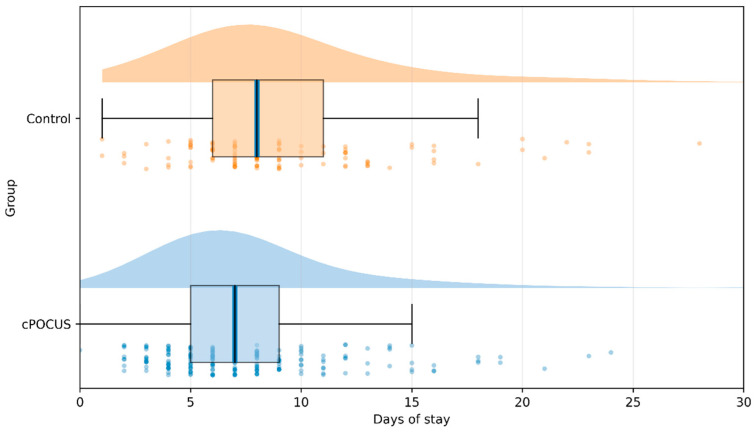
Comparison of length of hospital stay between the cardiac point-of-care ultrasound (cPOCUS) group and the control group. Raincloud plots of hospital length of stay in the cPOCUS and control groups. Half-violin plots represent kernel density, box plots show the median and interquartile range (IQR), and dots indicate individual patient observations. Median (IQR) length of stay was 7 days [4] in the cPOCUS group and 8 days [5] in the control group (*p* = 0.015). For visualization purposes, the *x*-axis is truncated at 30 days.

Length of stay was significantly longer in the control group compared with the cPOCUS group [8 days (IQR 5) vs. 7 days (IQR 4); *p* = 0.015], corresponding to an absolute median difference of 1 hospital day (Figure 2).

Echocardiographic findings among patients with available reports (n = 318) are summarized in Table 2. In the cPOCUS group (n = 207), the most frequent abnormalities were left ventricular hypertrophy (45.0%) and left atrial dilation (28.5%), followed by valvular regurgitation, including aortic insufficiency in 22.2% and mitral insufficiency in 20.2% of patients. Segmental wall motion abnormalities were observed in 14.0%, and intracavitary masses were infrequent (1.9%), with left atrial masses identified in 0.5% of cases. Mitral and aortic stenosis were detected in 6.8% and 6.3% of patients, respectively. In the control group (n = 111), left ventricular hypertrophy was also the most common finding (45.9%), followed by left atrial dilation (38.7%). Valvular insufficiency was present in 23.4% for the mitral valve and 17.1% for the aortic valve, whereas mitral and aortic stenosis were observed in 2.7% and 6.3% of patients, respectively. Segmental wall motion abnormalities were identified in 12.6% of patients, and intracavitary masses were detected in 1.8%, with no left atrial masses reported. Left ventricular systolic function was preserved in most control patients, with a normal ejection fraction in 91.0%, mild reduction in 6.3%, and severe reduction in 2.7%.

In multivariable linear regression analysis, evaluation with cPOCUS was independently associated with a shorter hospital length of stay [β coefficient (standard error): −1.49 (0.73)], after adjustment for potential confounding factors (Table 3). The magnitude of effect suggests an adjusted reduction of approximately 1.5 hospital days associated with cPOCUS evaluation.

## 4. Discussion

To our knowledge, this is the first study specifically designed to evaluate the association between neurologist-performed cPOCUS and hospital length of stay in an acute ischemic stroke population. The neurologist-performed cPOCUS was associated with a shorter length of stay among patients admitted to an SU, particularly in a setting with restricted access to non-urgent cardiac imaging techniques.

Echocardiography is a key component of etiological evaluation in ischemic stroke and has been associated with a lower likelihood of undetermined etiology and reduced in-hospital mortality [3]; however, access to this complementary test is frequently limited, which may delay diagnostic completion and subsequent therapeutic decision-making [12]. In this context, the use of focused cardiac ultrasound performed by non-cardiologists with appropriate training has been proposed as a strategy to optimize in-hospital workflows [2].

The clinical utility of cPOCUS performed by non-cardiologists has been evaluated in several studies in critically ill populations [13,14,15,16]. These studies have shown that cPOCUS is a feasible technique and an effective screening tool for ventricular function, with no relevant differences compared with formal transthoracic echocardiography (TTE) [15], thereby facilitating early management and clinical decision-making [16]. However, the objectives of cPOCUS in these settings differ from those in cerebrovascular disease, where the primary focus is the identification of potential embolic cardiac sources rather than comprehensive cardiac assessment.

In Spain, neurologist-performed cPOCUS has been implemented in a regulated manner since 2019 following accreditation by the Spanish Society of Cardiology [5]. Although its adoption has increased in SU, dissemination remains limited, and evidence regarding its overall clinical impact is still scarce [2]. Nevertheless, available studies report favorable results. Early experience with simplified echocardiographic protocols, such as the Stroke Echoscan, demonstrated accurate screening for major embolic sources by trained neurologists [4], and subsequent observational studies have confirmed the diagnostic utility of cPOCUS in routine stroke care [2,17]. In a prospective study, López-Dequidt et al. [18] reported improved detection of previously unrecognized structural heart disease, with potential implications for secondary prevention in patients at high risk of early recurrence.

The impact of cPOCUS on hospital length of stay has not been previously examined as a primary outcome in stroke populations. In the present study, implementation of neurologist-performed cPOCUS was associated with a median reduction of one hospital day. Although modest, this difference may be clinically meaningful, as shorter hospitalization has been associated with a lower risk of in-hospital complications and improved efficiency of stroke unit care [1]. In addition, previous studies using focused echocardiographic protocols in acute stroke settings have suggested that earlier etiological assessment may facilitate earlier discharge and more efficient resource utilization [4], potentially translating into reduced healthcare costs.

This study has several limitations. Its retrospective observational design may be associated with information bias and does not allow causal inference. In addition, during the study period, the availability of cPOCUS depended on the presence of an accredited neurosonologist, resulting in a non-random allocation of patients. Although schedule-based allocation partially reduces clinician-driven selection bias, it does not eliminate potential confounding related to admission timing, staffing, stroke severity patterns, or other organizational factors.

Operator-dependent variability is another relevant limitation. Although examinations were performed by trained neurologists using standardized protocols, formal inter-observer agreement was not assessed.

Prospective, multicenter studies with standardized implementation of cPOCUS are warranted to confirm these findings and better define its impact on clinical outcomes and healthcare resource utilization.

## 5. Conclusions

In this retrospective observational study, neurologist-performed cPOCUS was associated with shorter hospital length of stay among patients with acute ischemic stroke admitted to a Stroke Unit. While the findings suggest that cPOCUS may contribute to more efficient in-hospital workflows, the study design precludes causal inference. Prospective controlled studies are needed to determine whether systematic implementation of cPOCUS directly reduces length of stay and improves healthcare efficiency in stroke care pathways.

## Figures and Tables

**Table 1:** Baseline and hospitalization data.

	Total (n = 332)	cPOCUS Group * (n = 219)	Control Group ** (n = 113)	*p* Value
Demographics and comorbidities				
Female sex, n (%)	131 (39.6)	82 (37.6)	49 (43.4)	0.30
Age, median (IQR)	70 (20)	70 (19)	70 (21)	0.05
Hypertension, n (%)	192 (57.8)	131 (59.8)	61 (54)	0.30
Dyslipidemia, n (%)	139 (41.9)	102 (46.6)	37 (32.7)	0.02
Diabetes mellitus, n (%)	112 (33.7)	78 (35.6)	34 (30.1)	0.33
Ischemic heart disease, n (%)	23 (6.9)	18 (8.2)	5 (4.4)	0.19
Atrial fibrillation, n (%)	95 (28.6)	62 (28.3)	33 (29.2)	0.86
Smoking, n (%)	105 (31.6)	66 (30.1)	39 (34.5)	0.42
Alcoholism, n (%)	26 (7.8)	17 (7.8)	9 (8)	0.95
Drug use, n (%)	24 (7.2)	16 (7.3)	9 (7.96)	0.74
Vascular territory				
Anterior/posterior, n (%)	312 (94)/20 (6)	218 (94.4)/13 (5.6)	94 (93.1)/7(6.9)	0.69
NIHSS, median (IQR)	5 (9)	4.5 (7)	5 (11)	0.187
Reperfusion treatments				
Intravenous fibrinolysis, n (%)	59 (17.8)	46 (21)	22 (19.5)	0.26
Mechanical thrombectomy, n (%)	70 (21.1)	54 (24.7)	16 (14.2)	0.02
In-hospital complications				
Lower respiratory tract infection, n (%)	17 (5.1)	7 (3.2)	10 (8.8)	0.35
Urinary tract infection, n (%)	19 (5.7)	13 (5.9)	6 (5.3)	0.82

* cPOCUS group: Cardiac Point-of-Care Ultrasound (cPOCUS) with or without echocardiography. ** Control group: only echocardiography.

**Table 2:** Echocardiographic findings in cPOCUS and control group patients with available reports (n = 318).

	cPOCUS Group * (n = 207)	Control Group ** (n = 111)
Left atrial dilation, n (%)	59 (28.5)	43 (38.7)
Left ventricular dilation, n (%)	14 (6.8)	3 (2.7)
Left ventricular hypertrophy, n (%)	93 (45)	51 (45.9)
Segmental hypokinesia, n (%)	29 (14)	14 (12.6)
Intracavitary masses, n (%)	4 (1.9)	2 (1.8)
Left atrial masses, n (%)	1 (0.5)	0 (0)
Mitral insufficiency, n (%)	42 (20.2)	26 (23.4)
Aortic insufficiency, n (%)	46 (22.2)	19 (17.1)
Mitral stenosis, n (%)	14 (6.8)	3 (2.7)
Aortic stenosis, n (%)	13 (6.3)	7 (6.3)
Left Ventricular Ejection Fraction (LVEF)		
Normal (≥50%), n (%)	-	101 (91)
Mild reduction (30–50%), n (%)	-	7 (6.3)
Severe reduction (<30%), n (%)	-	3 (2.7)

* cPOCUS group: Cardiac Point-of-Care Ultrasound (cPOCUS) screening with or without echocardiography. ** Control group: only echocardiography.

**Table 3:** Multiple linear regression analysis of hospital length of stay.

Model	Unstandardized Coefficients *		*p*-Value	95% Confidence Interval for B	
	B	Standard Error		Lower Limit	Upper Limit
(Constant)	7.515	0.692	<0.01	6.152	8.878
cPOCUS group	−1.490	0.733	0.04	−2.933	−0.048
NIHSS	0.200	0.052	<0.01	0.097	0.303
Lower respiratory tract infection	9.467	1.574	<0.01	6.368	12.565
Posterior vascular territory	2.552	1.366	0.06	−0.137	5.240

* Stepwise multiple linear regression model adjusted for female sex, age, dyslipidemia, ischemic heart disease, atrial fibrillation, smoking, alcoholism, drug use, posterior circulation, NIHSS, mechanical thrombectomy, lower respiratory tract infection, and urinary tract infection. R^2^ = 0.223.

## Data Availability

The original contributions presented in this study are included in the article. Further inquiries can be directed to the corresponding author(s).
